# *Borreliella burgdorferi* factor H-binding proteins are not required for serum resistance and infection in mammals

**DOI:** 10.1128/iai.00529-23

**Published:** 2024-01-30

**Authors:** Nicholas A. Cramer, Kalya M. Socarras, Joshua Earl, Garth D. Ehrlich, Richard T. Marconi

**Affiliations:** 1 Department of Microbiology and Immunology, Virginia Commonwealth University Medical Center, Richmond, Virginia, USA; 2 Department of Microbiology, Drexel University College of Medicine, Philadelphia, Pennsylvania, USA; University of California, Davis, Davis, California, USA

**Keywords:** factor H, Lyme disease, CspA, CspZ, OspE, dogs, cp32, complement evasion

## Abstract

The causative agent of Lyme disease (LD), *Borreliella burgdorferi*, binds factor H (FH) and other complement regulatory proteins to its surface. *B. burgdorferi* B31 (type strain) encodes five FH-binding proteins (FHBPs): CspZ, CspA, and the OspE paralogs OspE_BBN38_, OspE_BBL39_, and OspE_BBP38_. This study assessed potential correlations between the production of individual FHBPs, FH-binding ability, and serum resistance using a panel of infectious *B. burgdorferi* clonal populations recovered from dogs. FHBP production was assessed in cultivated spirochetes and by antibody responses in naturally infected humans, dogs, and eastern coyotes (wild canids). FH binding specificity and sensitivity to dog and human serum were also assessed and compared. No correlation was observed between the production of individual FHBPs and FH binding with serum resistance, and CspA was determined to not be produced in animals. Notably, one or more clones isolated from dogs lacked CspZ or the OspE proteins (a finding confirmed by genome sequence determination) and did not bind FH derived from canines. The data presented do not support a correlation between FH binding and the production of individual FHBPs with serum resistance and infectivity. In addition, the limited number and polymorphic nature of cp32s in *B. burgdorferi* clone DRI85A that were identified through genome sequencing suggest no strict requirement for a defined set of these replicons for infectivity. This study reveals that the immune evasion mechanisms employed by *B. burgdorferi* are diverse, complex, and yet to be fully defined.

## INTRODUCTION

Lyme disease (LD) is the most common vector-borne disease in the northern hemisphere (1–4). *Borreliella burgdorferi*, the primary causative agent of LD in North America (5, 6), is maintained in an enzootic cycle involving *Ixodes* ticks and diverse vertebrate hosts (7). LD is a growing health threat, with more than 470,000 clinician-diagnosed human cases each year in the United States (1). While routine screening for LD in humans is not practiced, yearly screening is recommended in veterinary medicine for dogs residing in or near endemic regions (8). The Companion Animal Parasite Council (CAPC) reported 465,721 *B. burgdorferi* antibody-positive tests in client-owned dogs in 2023 (https://capcvet.org). However, since the CAPC collects data for only 30% of the total number of United States Department of Agriculture (USDA)-approved *B. burgdorferi* antibody tests run yearly in the US, the actual number of positive Ab tests likely exceeds 1 million. While wildlife and wild canids have a high exposure risk to tick-transmitted pathogens (9), little is known regarding the impact of tick-borne diseases on wildlife health. A serological analysis of plasma samples from eastern coyotes (*Canis lupus*) from the Commonwealth of Pennsylvania collected from 2015 to 2018 revealed that 64.8% and 72.7% were antibody-positive for *B. burgdorferi* and *Anaplasma phagocytophilum*, respectively (10).

The LD spirochetes must evade innate immunity to establish an infection in mammals. The mechanisms associated with complement evasion have been the subject of intensive research [reviewed in reference (11)]. Negative regulators of complement, including factor H (FH), FH-like protein 1, FH-related proteins, and C1r- and C4b-binding proteins, have been demonstrated to bind to specific *Borreliella* surface proteins (12–16). *B. burgdorferi* B31 produces five FH-binding proteins: CspZ, CspA, and the OspE paralogs OspE_BBN38_, OspE_BBL39_, and OspE_BBP38_. All are encoded by genes carried by plasmids or 32 kb prophage (cp32s) (17). The molecular interactions between recombinant FH-binding proteins (FHBPs) and FH are well characterized (18–22). While *in vitro* studies have revealed that FH bound to the surface of *B. burgdorferi* is competent to serve as a cofactor for factor I-mediated cleavage of C3b, an important opsonin [reviewed in reference (23)], the significance of FH binding in ticks and mammals remains an open question. In this study, we assessed 24 clonal populations of *B. burgdorferi* that were recovered from infected dogs for FHBP production, FH binding, and serum sensitivity. We demonstrate that CspA is not produced during infection in mammals and that the OspE and CspZ proteins (and FH binding in general) are not required for serum resistance. The data suggest that complement evasion may be mediated through FH-independent mechanisms.

## MATERIALS AND METHODS

### Bacterial strains and growth conditions

*B. burgdorferi* clones analyzed in this report were recovered from tissue biopsies from dogs that were experimentally infected using *Ixodes scapularis* or *Ixodes pacificus* ticks (24). The original non-clonal isolates were assigned the prefixes DRI, DCT, DWI, or DCA to indicate that they originated from dogs (D) that were infected using ticks collected in Rhode Island (RI), Connecticut (CT), Wisconsin (WI), or California (CA). An arbitrary identifier number follows these designations. A letter designation further differentiates the clones obtained by sub-surface plating. All clones were cultivated in BSK-H complete media supplemented with 6% rabbit serum at 34°C, and growth was monitored using wet mounts and dark-field microscopy. Cells were harvested by centrifugation, and cell lysates were generated for SDS-PAGE and immunoblot analyses, as described below. *B. burgdorferi* B31 (clone 5A4) was a control, as its genome sequence is known (17), and its FHBPs have been well characterized (25).

### Serum samples

Serum samples from *B. burgdorferi* antibody-positive client-owned dogs were provided by the College of Veterinary Medicine, North Carolina State University. Blood samples collected from eastern coyotes (*Canis lupus*) harvested in PA between 2015 and 2020 were obtained under a Pennsylvania Game Commission Special Use Scientific Studies Permit (#48548). The coyote serum had been previously assessed for antibodies to *B. burgdorferi, A. phagocytophilum* (10)*,* canine parvovirus, and distemper virus (26). The coyote samples were included in this study as they are wild canids with high exposure risk for tick-borne diseases. Their analysis is not complicated by previous vaccination or acaricide applications. Human serum samples were obtained from the Lyme disease Biobank (27). Note that except for negative control sera, all serum samples used in this study were confirmed to be antibody positive for *B. burgdorferi* by whole-cell lysate immunoblot and enzyme-linked immunosorbent assay (ELISA) using recombinant *B. burgdorferi* VlsE and other characterized proteins as the detection antigens.

### Protein nomenclature and generation of recombinant proteins

Several nomenclature strategies have been used to differentiate the FHBPs of *B. burgdorferi* and related species. These proteins have been collectively referred to as complement regulator-acquiring surface proteins (28), Erps (OspEF-related proteins) (29), or Elps (30). Erp designations have been broadly applied to diverse and functionally distinct proteins belonging to different gene families (31). For example, the OspF proteins (BB039, BBM38, and BBR42), which do not bind to complement regulatory proteins (32), have been referred to as Erps in some studies. To be consistent with previous studies from our laboratory (19, 33–36), we employ the original protein and open reading frame (ORF) designations assigned to the FHBPs of *B. burgdorferi* B31 (CspA, CspZ, OspE_BBL39_, OspE_BBP38_, and OspE_BBN38_). OspE_BBL39_ and OspE_BBP38_ are encoded by genes located on different cp32s but are identical in sequence, and hence, we collectively refer to them as OspE_BBL39/P38_ (17).

Genes encoding recombinant proteins were PCR amplified using *B. burgdorferi* B31 DNA and standard conditions with Phusion polymerase (ThermoScientific). PCR primers were synthesized with restriction sites or ligase-independent cloning tails to allow for cloning into pET45b(+) and pET46, respectively (Novagen), as previously described (37). Some genes were codon optimized for expression in *Escherichia coli*, synthesized (Genscript), and provided by the supplier directly in pET45b(+). Recombinant plasmids were transformed into *E. coli* BL21/DE3 cells and protein production induced using isopropyl β-D-1-thiogalactopyranoside (IPTG) (0.1 mM) or autoinduction (overnight). The cells were harvested by centrifugation, lysed with a high-pressure cell homogenizer, and purified via their N-terminal hexa-His tags from the soluble fraction using nickel affinity chromatography on an AKTA FPLC purification platform (Cytiva). Protein purification was performed as previously described (38).

### Generation of antisera

Antisera to recombinant proteins were generated in Sprague-Dawley rats (10). In brief, rats were anesthetized with isoflurane, injected intraperitoneally with 40 µg of each recombinant protein in Freund’s complete adjuvant (day 0), and then boosted with 40 µg of protein in Freund’s incomplete adjuvant (days 21 and 35). On day 42, the rats were euthanized, blood was collected by cardiac puncture, and serum was harvested using standard methods. All animal experiments were conducted following the Guide for the Care and Use of Laboratory Animals (eighth edition) and in accordance with protocols peer-reviewed and approved by Virginia Commonwealth University Institutional Animal Care and Use Committees.

### SDS-PAGE and immunoblot analyses

Cell lysates were fractionated by SDS-PAGE in 15% Criterion AnyKDa Gels (Bio-Rad). The proteins were transferred to polyvinylidene fluoride (PVDF) membranes (0.22 µm) using the Trans-Blot Turbo system according to manufacturer instructions (Bio-Rad). The membranes were incubated with blocking solution (5% non-fat dried milk, 0.2% Tween-20 in phosphate buffered saline [PBS]; 1 h; room temperature) followed by the addition of anti-CspA (1:1,000), anti-CspZ (1:1,000), anti-OspE_BBL39/P39_ (1:20,000), or anti-OspE_BBN38_ antisera (1:1,000). The antisera were removed. The blots were washed three times (0.2% Tween-20 in PBS), followed by the addition of the appropriate secondary antibodies (1:40,000 in blocking solution). Bound IgG was detected using Clarity Western ECL substrate (5 min; Bio-Rad). Images were captured using a ChemiDoc Touch imaging system (Bio-Rad; auto-optimal settings). Images were cropped for presentation purposes.

### FH-binding assays

FH binding was assessed as previously described (25). In brief, whole-cell lysate immunoblots were blocked, washed, and incubated with 10% dog, coyote, or human serum (human serum from Complement Tech). Bound FH was detected using sheep anti-FH antiserum (1:1,000; Invitrogen) with rabbit anti-sheep IgG as the secondary (Pierce). Bound IgG was detected as above.

### ELISA

ELISA plate wells were coated with 500 ng of recombinant protein in bicarbonate buffer (overnight; 4°C) (39). Dog, coyote, human, or mouse sera were added (1:1,000 dilution; 1 h; room temperature), followed by three washes. A secondary anti-IgG antibody was added at a 1:15,000 dilution. After washing, the 2,2'-azino-bis[3-ethylbenzothiazoline-6-sulfonic acid (ABTS)] substrate was added (20 min), and absorbance was measured at 405 nm. Immobilized bovine serum albumin (BSA) served as an immobilized protein control for non-specific antibody binding. Serum samples were scored as antibody positive if the mean absorbance value was twofold greater than the mean absorbance of the serum with BSA or preimmune serum.

### Serum sensitivity analyses

Mid-log phase *B. burgdorferi* B31 cells were incubated (34°C; 18 h) in 0%, 40%, and 80% (final concentrations) dog or human complement-preserved serum (Innovative Research). The assays were performed in triplicate. Live cells were counted (400×; an average of five fields of view) using dark-field microscopy. Percent killing was calculated by dividing the number of live cells in each tube by the number of live cells in the controls. All statistical analyses were conducted as previously described (40).

### Mouse infectivity analyses

C3H/HeN mice were injected (subcutaneously) with 10^5^ mid-log phase *B. burgdorferi* B31 or *B. burgdorferi* DRI85A and sacrificed 17 days later. Ear tissue and urinary bladders were collected, macerated, and placed in media with a standard *Borreliella* antibiotic cocktail (41). Blood was harvested from each mouse by cardiac puncture. Seroconversion was assessed by ELISA, as detailed above, using the infection serum at a 1:1,000 dilution.

### DNA extraction, preparation, and sequencing

Bacterial pellets were resuspended in 200 µL of 1× PBS and transferred to a 2-mL bead beating tube (Matrix E; MP Biomedicals). Proteinase K (20 µL; Qiagen) was added, and the cells homogenized (SPEX 1600 MiniG; 1 min; 1,500 Hz Fisher Scientific). DNA was extracted using the Qiagen DNeasy Blood & Tissue Kit according to the manufacturer’s instructions (Qiagen). The DNA was quantified using the 1× dsDNA HS kit (ThermoFisher Scientific on a Qubit). DNA was prepped with the SMRTbell Template Prep Kit 2.0 (Pacific Biosciences) to make PacBio SMRTbell libraries with barcodes sourced from the Barcoded Overhang Adaptor Kit 8A and 8B (Pacific Biosciences). The sequencing primers were annealed and bound to Polymerase 3.0 using the Sequel Binding Kit 3.0 (Pacific Biosciences). The bound complex was purified and sequenced on a PacBio Sequel I using an SMRT Cell M1 v3 tray (Pacific Biosciences). The spike-in controls for each PacBio Sequel I run were from the Internal Control Kit 3.0 (Pacific Biosciences).

### Genome assembly, annotation, and pangenome construction

Genome sequences were processed using pbcromwell (v1.0.4) (Pacific Biosciences) and the barcoded data were demultiplexed using pb_demux_subreads and assembled using pb_assembly_microbial. The base modification motifs for each assembly were computed using pb_basemods. The species’ identity was verified using the taxator and GTDB-Tk (v1.7.0) with refpack (v r202). All reference and sequenced *Borreliella* assemblies were annotated with Prokka (v 1.11) (42) and EggNOG (43), and then homologous genes were clustered with Roary (v 3.5.1) (44). The BlastP threshold was calculated consecutively at 5% intervals to determine the final threshold (75%).

## RESULTS

### Comparative analysis of the *in vitro* expression of FHBPs by *B*. *burgdorferi* clonal populations

**Fig 1:**
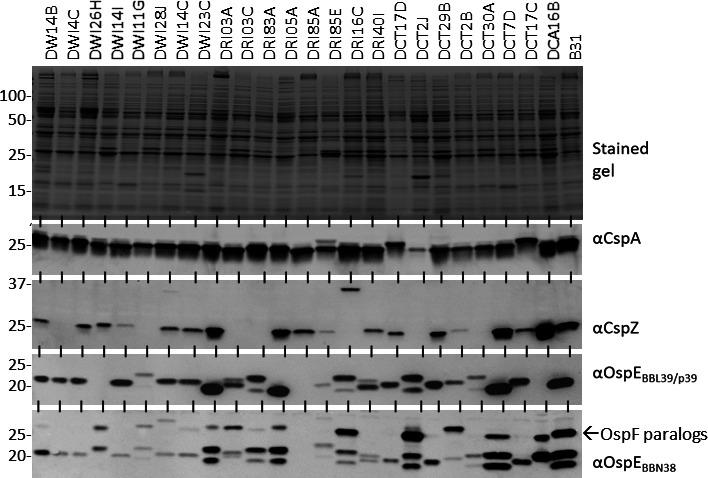
FHBP production by *B. burgdorferi* clones derived from infected dogs. Cell lysates of *B. burgdorferi* clonal populations were fractionated by SDS-PAGE using AnyKda gels. Clone designations are indicated along the top of the figure. *B. burgdorferi* B31 served as a positive control. One gel was stained with Coomassie brilliant blue to demonstrate similar loading (top panel). Immunoblots were generated and screened with antisera to the FHBPs (indicated to the right). The molecular weight (MW) of OspE_BBN38_ and OspE_BBL39_ are 20.675 and 19.560 kDa, respectively. Variation in the MW of the OspE paralogs in some clones rendered definitive identification at the paralog level difficult. Hence, the proteins are labeled simply as OspE. Note that anti-OspE_BBN38_ also reacts with OspF (indicated), which is not an FHBP. The migration position of MW standards is indicated to the left. The antisera were used at a 1:1,000 dilution. All methods were as described in the text, and the images were cropped for presentation purposes.

FHBP profiles of low-passage *B. burgdorferi* clonal populations isolated from infected dogs were determined by immunoblot analysis of cell lysates using anti-OspE_BBN38_, anti-OspE_BBL39/P38_ anti-CspA, and anti-CspZ antisera (Fig. 1). Anti-OspE_BBL39/P38_ antisera and anti-OspE_BBN38_ antisera reacted with proteins of the predicted molecular weight in most clones except *B. burgdorferi* DRI85A. Note that anti-OspE_BBN38_ antibodies bound to one or more of the OspF proteins (26–28 kDa; indicated in Fig. 1) produced by some clones. The absence of immunoreactive OspE and OspF proteins in DRI85A suggests that this clone lacks one or more cp32s that carry these genes (discussed in detail below). Consistent with the universal distribution of linear plasmid 54 (lp54), which carries *cspA*, a protein consistent in size with CspA was detected in all clones. In contrast, CspZ was detected in only 17 of 24 clones. This observation is consistent with previous studies that demonstrated that the *cspZ* encoding plasmid (lp28-3) is polymorphic and (45) not carried by all isolates (46).

### FH-binding analyses

**Fig 2:**
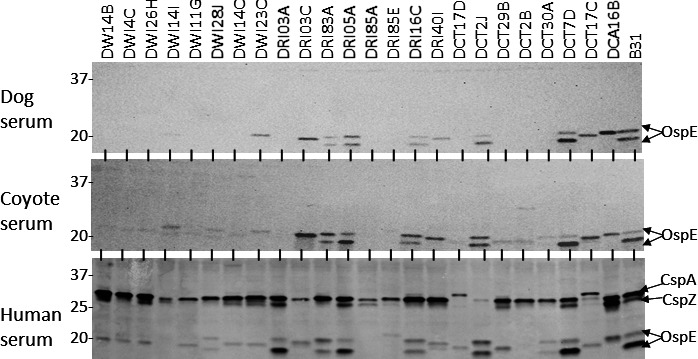
Comparative analysis of the FHBP profiles of *B. burgdorferi* clonal populations. Immunoblots of cell lysates were generated as described in the Materials and Methods section and in Fig. 1. The membranes were incubated with the dog, eastern coyote, or human sera as indicated. The sera, which served as the species-specific FH source, were confirmed to be antibody-negative for *B. burgdorferi* before use. Bound FH was detected using anti-FH antiserum, as detailed in the text. Clone designations are indicated along the top of the figure. The identities of the proteins that bound FH are indicated to the right.

To determine if the FHBPs of the *B. burgdorferi* clones bind FH from humans, dogs, and eastern coyotes, whole-cell lysate immunoblots were incubated with serum from each (Fig. 2). Human FH bound to CspZ, CspA, and one or more OspE paralogs in most clones. In contrast, dog and coyote FH bound only to OspE proteins in a subset of clones. Canine FH did not bind to proteins in the OspE size range in clone DRI85A. This is consistent with the lack of detectable OspE proteins in the immunoblot analyses described above. The fact that clone DRI85A does not produce proteins that bind FH is noteworthy, as this clone was derived from an infected dog and, as shown below, is infectious in mice.

### Antibody responses to the FHBPs in *B*. *burgdorferi* antibody-positive dogs, coyotes, and humans

To determine if OspE_BBL39/P38_, OspE_BBN38_, CspZ, and CspA were produced in naturally infected humans, dogs, and eastern coyotes, we screened serum from *B. burgdorferi* antibody-positive animals against recombinant FHBPs. Antibodies to OspE_BBN38_ and anti-OspE_BBL39/P38_ were detected in 42% and 6% of human serum samples, respectively (Table 1). Antibodies to CspZ and CspA were detected in 14 of the 100 of the dog and coyote serum samples. While the absence of detectable antibodies to the FHBPs in most serum samples suggests that they are not universally produced during infection in humans and canines, we cannot exclude the possibility that differences in immune responses among animals, varying surface exposure or expression levels, are contributing factors.

**TABLE 1:** Percentage of human, dog, and coyote serum samples positive for *Borreliella* proteins 610 by ELISA screening

Detection antigen	Human (*N* = 50)	Dog (*N* = 50)	Coyote (*N* = 50)
CspA	00.0%	00.0%	02.0%
CspZ	00.0%	00.0%	14.0%
OspE_BBN38_	42.0%	34.0%	12.0%
OspE_BBL39/P38_	06.0%	38.0%	24.0%
OspE_BBN38_ or OspE_BBL39/P38_	46.0%	48.0%	28.0%
VlsE	100.0%	100.0%	100.0%
BSA	00.0%	00.0%	00.0%

### Serum sensitivity to human and canine serum

The sensitivity of 10 *B. burgdorferi* clones to increasing concentrations of complement-certified dog and human serum was assessed. Coyote serum was not tested since it is not commercially available at the “complement certified” grade. The percent killing among clones treated with 40% dog serum ranged from 11.4% to 100.0% (Table 2). The near complete killing of all clones (94.9% to 100.0%) was observed when exposed to 80% dog serum. The difference in percent killing of DWI14C and DWI14I in 40% canine serum (11.4% versus 100.0%) is noteworthy as these clones were isolated from the same tissue biopsy (24), and both produce CspA, CspZ, and at least one OspE protein. However, as detailed above, canine FH does not bind to CspA or CspZ. When the clones were treated with 40% and 80% human serum, cell killing ranged from 0.0% to 95.6% and 64.1% to 100.0% killing, respectively. Clones DRI85A and DRI03A were not killed by 40% human serum, whereas clones DWII4I and DWII4C were highly sensitive (>92%). Cells incubated in media alone or 80% heat-inactivated (HI) serum served as controls, and as expected, HI serum did not negatively impact growth. The fact that the CspA protein produced by all clones bound human FH but did not convey serum resistance indicates that CspA in and of itself is not sufficient to protect against human complement.

**TABLE 2:** Sensitivity of diverse *B. burgdorferi* clones to canine and human serum

	BSK H media	Heat inactivated 80% serum	40% serum (complement certified)	80% serum (complement certified)
Canine serum assays				
B31	0.0 ± 0.0	0.0 ± 0.0	72.1 ± 0.5	100.0 ± 0.0
DWI14I	0.0 ± 0.0	0.0 ± 0.0	100.0 ± 0.0	100.0 ± 0.0
DWI28J	0.0 ± 0.0	0.0 ± 0.0	39.2 ± 4.6	94.9 ± 0.6
DWI11G	0.0 ± 0.0	0.0 ± 0.0	100.0 ± 0.0	100.0 ± 0.0
DWI14C	0.0 ± 0.0	0.0 ± 0.0	11.4 ± 4.3	100.0 ± 0.0
DCT29B	0.0 ± 0.0	0.0 ± 0.0	91.8 ± 1.5	100.0 ± 0.0
DCT2J	0.0 ± 0.0	0.0 ± 0.0	93.8 ± 2.1	100.0 ± 0.0
DRI85A	0.0 ± 0.0	0.0 ± 0.0	37.8 ± 3.5	100.0 ± 0.0
DRI03A	0.0 ± 0.0	0.0 ± 0.0	78.9 ± 1.5	96.8 ± 1.1
DRI40I	0.0 ± 0.0	0.0 ± 0.0	100.0 ± 0.0	100.0 ± 0.0
Human serum assays				
B31	0.0 ± 0.0	0.0 ± 0.0	34.1 ± 1.3	100.0 ± 0.0
DWI14I	0.0 ± 0.0	0.0 ± 0.0	92.6 ± 0.6	100.0 ± 0.0
DWI28J	0.0 ± 0.0	0.0 ± 0.0	89.5 ± 2.5	90.0 ± 1.3
DWI11G	0.0 ± 0.0	0.0 ± 0.0	95.6 ± 1.1	95.6 ± 0.8
DWI14C	0.0 ± 0.0	0.0 ± 0.0	69.8 ± 2.5	88.1 ± 2.6
DCT29B	0.0 ± 0.0	0.0 ± 0.0	19.1 ± 1.5	72.5 ± 3.2
DCT2J	0.0 ± 0.0	0.0 ± 0.0	77.8 ± 3.2	96.1 ± 0.6
DRI85A	0.0 ± 0.0	0.0 ± 0.0	0.0 ± 0.0	64.1 ± 4.7
DRI03A	0.0 ± 0.0	0.0 ± 0.0	0.0 ± 0.0	75.7 ± 5.5
DRI40I	0.0 ± 0.0	0.0 ± 0.0	68.2 ± 1.5	100.0 ± 0.0

### Infectivity analysis of *B*. *burgdorferi* clone DRI85A

**Fig 3:**
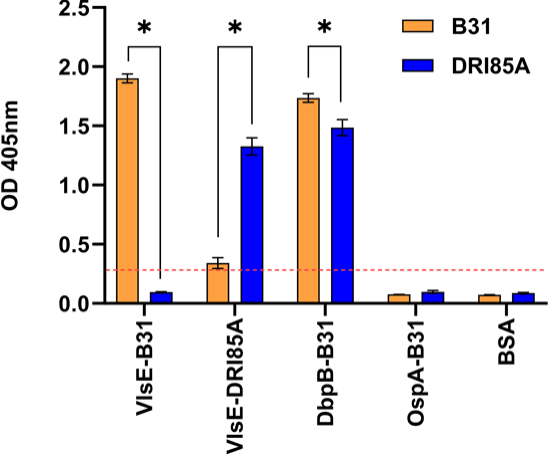
Immunoblot analysis and ELISA of *B. burgdorferi* clones DRI85A and B31 using serum from infected mice. Mice were inoculated with *B. burgdorferi* clone DRI85A or *B. burgdorferi* B31. Seventeen days post-inoculation, blood was collected, and the serum was harvested. To assess seroconversion, the sera were screened by ELISA against recombinant DbpB and VlsE (B31 sequence) and the VlsE protein derived from clone DRI85A. OspA, a *Borreliella* protein that is not expressed during infection, and BSA, served as negative controls. Significance was assessed as detailed in the text (* indicates *P* < 0.05).

The aforementioned data suggest that FHBPs are not required for infectivity. To assess this directly, the ability of clone DRI85A to infect mice was tested with B31 serving as a positive control. DRI85A was cultured from ear biopsies and/or the bladder of four of six mice. Seroconversion was assessed by ELISA using DbpB, VlsE, and OspA as detection antigens (Fig. 3). Due to limited amino acid sequence identity (54%) between VlsE from DRI85A and B31, recombinant proteins were generated for both sequence variants and included in the ELISA. Serum from B31 and DRI85A infected mice displayed similar antibody responses to DbpB. However, the reaction to the VlsE proteins was variant-specific. Antibodies to the negative control proteins (OspA and BSA) were not detected. It can be concluded from the infectivity analyses that the OspE proteins and FH binding, in general, are not essential for the *Borreliella* infection of mice and dogs overall.

### Genome data

The genome of DRI85A is 1,482,443 bases and consists of 18 plasmids and a linear chromosome of 919,128 bases. The complete genome sequence of DRI85A and other clones analyzed in this study were determined as part of a large-scale pangenome project (Bioproject PRJNA1026537) that will be described separately. Here, we present only information relevant to FH binding and immune evasion. *B. burgdorferi* DRI85A carries only four cp32-related replicons, none encoding *ospE* or *ospF* genes. By comparison, *B. burgdorferi* B31 carries 11 cp32s. The DRI85A cp32-related replicons consist of a 22,696 bp circular plasmid (contig 12) with 99.92% identity (100% query coverage) to cp32-4 of *B. burgdorferi* strain 297; a circular plasmid of 66,968 bp (contig 17) that is an imperfect “concatemer” of two cp32s; a 30,636 bp circular plasmid that shares 98.86% nt identity (100% query coverage) with cp32-6 from *B. burgdorferi* 297; and a 10,740 bp circular plasmid with 99.23% nt identity (98% query coverage) to a segment of cp32-8 of *B. burgdorferi* strain JD1. The absence of cp32s with *ospE* and *ospF* genes is consistent with the lack of detection of these proteins in the immunoblot and FH-binding analyses detailed above.

## DISCUSSION

The relative contribution of individual *B. burgdorferi* FHBPs in complement evasion has been challenging to determine due to functional redundancy and the variability of some replicons that encode the FHBPs. This report focused on our analysis of FH binding and serum sensitivity, exclusively on low-passage *B. burgdorferi* clonal populations recovered from dogs that were infected using field-collected ticks (24). This approach eliminates the variables associated with using non-clonal and lab-attenuated strains.

The FHBP profile of each clone was determined by screening whole-cell lysate immunoblots with antiserum generated against each *B. burgdorferi* FHBP. Consistent with a recent comprehensive genome analysis that reported that plasmid lp54 is universal (47), CspA was detected in all clones. Although CspA is expressed during cultivation, anti-CspA antibodies to CspA were not detected in any human or canine serum samples (*n* = 100). Earlier studies demonstrated transcriptional downregulation of *cspA* by *B. burgdorferi* when cultivated in media supplemented with human blood (48) or when cultured in dialysis membrane chambers implanted in the peritoneal cavity of rats (49). In addition, *cspA* mRNA was not detected in spirochetes residing in the skin of infected mice (50), and CspA was not detected in the tissues of infected mice using advanced proteomic approaches (51). Collectively, data presented to date firmly indicate that natural isolates do not produce CspA during infection in mammals. CspA may facilitate complement evasion in the mid-gut of fed ticks. In support of this possibility, *B. burgdorferi cspA* gene deletion mutants are rapidly cleared from the midgut of feeding nymphal ticks (52).

In contrast to CspA, CspZ was only detected in a subset of clones (17 of 24). It has been demonstrated that the plasmid that encodes CspZ is not universal among LD isolates (47). Analyses of *cspZ* expression during infection have led to different conclusions. This study detected anti-CspZ antibodies in only 14 canine (wild and domestic) sera and none of the human sera. In contrast, Baum et al. reported detecting anti-CspZ antibodies in laboratory-infected purpose-bred beagles (53). Dulebohn et al. experimentally cured lp28-3 from *B. burgdorferi* and then compared the ability of the mutated strain and its isogenic parental strain to infect mice, disseminate, and be taken up by ticks (acquisition) (54). The lp28-3-deficient strain disseminated in mice, but its acquisition by feeding ticks was inefficient, suggesting that proteins encoded by lp28-3 increase fitness. Coleman et al. directly tested the potential requirement for CspZ by deleting the gene and testing the mutant for its ability to infect mice (55). The *cspZ* deletion mutant was fully infectious in mice. The data presented here, the non-universal distribution of lp28-3, and gene deletion studies (cited above) indicate that CspZ is not required for complement evasion or infection of mammals and ticks.

The immunoblot analyses revealed that all clones cultivated *in vitro*, except DRI85A, produced one or more OspE proteins. Significant variation in the MW and number of OspE paralogs among clones was noted. ELISAs were conducted to indirectly assess expression *in vivo*. Antibodies to one or more OspE proteins were detected in 46%, 24%, and 50% of the human, dog, and coyote serum. The apparent absence of OspE and OspF proteins, encoded by cp32s, prompted us to determine the genome sequence of DRI85A. The genome sequence was determined using PacBio sequencing. The atypical cp32 content of DRI85A is described above. In that, DRI85A is a natural and non-genetically manipulated strain, it can be concluded that not all cp32s, including those that carry *ospE* and *ospF* genes, are not required for a strain to circulate in nature.

Many FHBPs selectively bind FH derived from different mammalian species (11). Since all clones analyzed in this study were recovered from dogs, we sought to determine if they display enhanced or specific binding to canine FH. Serum from dogs and eastern coyotes served as the FH source, with human serum as the comparator. Surprisingly, the CspZ and CspA proteins did not bind to canine FH and only weakly bound to some OspE proteins. In contrast, human FH is bound to CspA, CspZ, and most OspE proteins. The lack of binding of canine FH to CspA and CspZ suggests that these proteins do not contribute to FH-mediated complement evasion in dogs and, most likely, in other mammals.

Based on variable FHBP protein profiles, we speculated that clonal populations may differ in their sensitivity to dog and human complement. The serum sensitivity of 10 clones to complement-certified dog and human serum (0%, 40%, and 80%) was assessed. As detailed in the Results section and Table 2, serum sensitivity differed significantly among clones. A matching set of cells was incubated with the heat-inactivated serum to determine if the killing was complement-dependent. All clones tested were unaffected by exposure to 80% heat-inactivated serum, indicating that the serum-associated killing was due to complement. A correlation between FHBP expression (inferred from the immunoblots; Fig. 1) and FH binding (overlay assays; Fig. 2) with serum sensitivity/resistance was not observed. Previous studies have classified *B. burgdorferi*, *Borreliella afzelii*, and *Borreliella garinii* (the three primary causative agents of LD) as having high, intermediate, or low resistance to complement (56). However, as demonstrated here, serum sensitivity varies significantly among clones of a given species. Hence, we suggest that generalizations about serum resistance at the species level (23) are invalid and that serum sensitivity must be assessed at the clonal level.

The ability of DRI85A to survive in human and canine serum suggests that *ospE* and *ospF*, as well as most of the cp32s, are not required for infection. While DRI85A was recovered from an infected dog and is a low passage, it is possible that cp32s could have been lost during post-isolation cultivation. Its ability to infect mice was tested to verify the infectious phenotype of DRI85A. Spirochetes (10^5^) were delivered by needle injection, and 17 days later, the mice were euthanized, and biopsies and blood were collected. The biopsies were placed in media with antibiotics in an attempt to recover spirochetes. Clone DRI85A was cultured from the ear and/or bladder of four of six mice and elicited seroconversion against recombinant VlsE and DbpB. Notably, the antibody response in mice infected with B31 and DRI85A to VlsE variants was strain-specific. Sequence divergence between the two VlsE variants is found throughout the protein, including the C6 domain. A polypeptide corresponding to the C6 region is widely used as a Lyme disease diagnostic peptide (57). Like our findings, Baum et al. (58) also reported on the variant-specific immune responses to VlsE. These findings have implications regarding the reliability of the C6 peptide in diagnosing infection with, or exposure to, *Borreliella* species.

In summary, the analyses conducted in this study do not support a correlation between the production of FHBPs and FH binding or serum resistance. In addition, the limited and polymorphic nature of the cp32s in DRI85A indicates no strict requirement for a defined set of these replicons for infectivity. This conclusion is supported by a recent study by Wachter et al. in which a B31-derived clone that was experimentally cured of all cp32s, infected mice, and ticks (59). The results of this study call into question assumptions that we and others have made about the biological functions of CspZ, OspE, and the OspF proteins, their potential utility as vaccine candidates, and the role of cp32-encoded proteins in the enzootic cycle. Lastly, it is likely that there are complement evasion mechanisms employed by the LD spirochetes that are yet to be defined.

## References

[1] Nelson CA, Saha S, Kugeler KJ, Delorey MJ, Shankar MB, Hinckley AF, Mead PS. 2015. Incidence of clinician-diagnosed lyme disease, United States, 2005-2010. Emerg Infect Dis 21:1625–1631. doi:10.3201/eid2109.150417 26291194 PMC4550147

[2] Sykes RA, Makiello P. 2017. An estimate of Lyme borreliosis incidence in Western Europe. J Public Health (Oxf) 39:74–81. doi:10.1093/pubmed/fdw017 26966194

[3] Heyman P, Cochez C, Hofhuis A, van der Giessen J, Sprong H, Porter SR, Losson B, Saegerman C, Donoso-Mantke O, Niedrig M, Papa A. 2010. A clear and present danger: tick-borne diseases in Europe. Expert Rev Anti Infect Ther 8:33–50. doi:10.1586/eri.09.118 20014900

[4] Eisen RJ, Kugeler KJ, Eisen L, Beard CB, Paddock CD. 2017. Tick-borne zonoses in the United States: persistent and emerging threats to human health. ILAR J 58:319–335. doi:10.1093/ilar/ilx005 28369515 PMC5610605

[5] Benach JL, Bosler EM, Hanrahan JP, Coleman JL, Habicht GS, Bast TF, Cameron DJ, Ziegler JL, Barbour AG, Burgdorfer W, Edelman R, Kaslow RA. 1983. Spirochetes isolated from the blood of two patients with Lyme disease. N Engl J Med 308:740–742. doi:10.1056/NEJM198303313081302 6828119

[6] Burgdorfer W, Barbour AG, Hayes SF, Benach JL, Grunwaldt E, Davis JP. 1982. Lyme disease-a tick-borne spirochetosis?. Science 216:1317–1319. doi:10.1126/science.7043737 7043737

[7] Bissett ML, Hill W. 1987. Characterization of Borrelia burgdorferi strains from Ixodes pacificus ticks in California. J Clin Microbiol 25:2296–2301. doi:10.1128/jcm.25.12.2296-2301.1987 3323225 PMC269474

[8] Littman MP, Gerber B, Goldstein RE, Labato MA, Lappin MR, Moore GE. 2018. ACVIM consensus update on Lyme borreliosis in dogs and cats. J Vet Intern Med 32:887–903. doi:10.1111/jvim.15085 29566442 PMC5980284

[9] Tsao JI, Hamer SA, Han S, Sidge JL, Hickling GJ. 2021. The contribution of wildlife hosts to the rise of ticks and tick-borne diseases in North America. J Med Entomol 58:1565–1587. doi:10.1093/jme/tjab047 33885784

[10] Izac JR, Camire AC, Schuler EJA, Hatke AL, O’Bier NS, Oliver LD, Corondi A, Plocinski OC, Desmond RP, Naimi WA, Carlyon JA, Van Why KR, Shelly J, Marconi RT. 2020. Serologic evidence for the exposure of eastern coyotes (Canis latrans) in pennsylvania to the tick-borne pathogens Borreliella burgdorferi and Anaplasma phagocytophilum. mSphere 5:e00544-20. doi:10.1128/mSphere.00544-20 32817454 PMC7426170

[11] Kraiczy P. 2016. Travelling between two worlds: complement as a gatekeeper for an expanded host range of Lyme disease spirochetes. Vet Sci 3:12. doi:10.3390/vetsci3020012 29056721 PMC5644625

[12] Locke JW. 2019. Complement evasion in Borrelia spirochetes: mechanisms and opportunities for intervention. Antibiotics (Basel) 8:80. doi:10.3390/antibiotics8020080 31200570 PMC6627623

[13] Dulipati V, Meri S, Panelius J. 2020. Complement evasion strategies of Borrelia burgdorferi sensu lato. FEBS Lett 594:2645–2656. doi:10.1002/1873-3468.13894 32748966

[14] Lin YP, Frye AM, Nowak TA, Kraiczy P. 2020. New insights into CRASP-mediated complement evasion in the Lyme disease enzootic cycle. Front Cell Infect Microbiol 10:1. doi:10.3389/fcimb.2020.00001 32083019 PMC7002432

[15] Skare JT, Garcia BL. 2020. Complement evasion by Lyme disease spirochetes. Trends Microbiol 28:889–899. doi:10.1016/j.tim.2020.05.004 32482556 PMC7572514

[16] Garrigues RJ, Powell-Pierce AD, Hammel M, Skare JT, Garcia BL. 2021. A structural basis for inhibition of the complement initiator protease C1R by Lyme disease spirochetes. J Immunol 207:2856–2867. doi:10.4049/jimmunol.2100815 34759015 PMC8612984

[17] Fraser CM, Casjens S, Huang WM, Sutton GG, Clayton R, Lathigra R, White O, Ketchum KA, Dodson R, Hickey EK, et al.. 1997. Genomic sequence of a Lyme disease spirochaete, Borrelia burgdorferi. Nature 390:580–586. doi:10.1038/37551 9403685

[18] Alitalo A, Meri T, Chen T, Lankinen H, Cheng Z-Z, Jokiranta TS, Seppälä IJT, Lahdenne P, Hefty PS, Akins DR, Meri S. 2004. Lysine-dependent multipoint binding of the Borrelia burgdorferi virulence factor outer surface protein E to the C terminus of factor H. J Immunol 172:6195–6201. doi:10.4049/jimmunol.172.10.6195 15128807

[19] McDowell JV, Wolfgang J, Senty L, Sundy CM, Noto MJ, Marconi RT. 2004. Demonstration of the involvement of outer surface protein E coiled coil structural domains and higher order structural elements in the binding of infection-induced antibody and the complement-regulatory protein, factor H. J Immunol 173:7471–7480. doi:10.4049/jimmunol.173.12.7471 15585873

[20] Cordes FS, Roversi P, Kraiczy P, Simon MM, Brade V, Jahraus O, Wallis R, Skerka C, Zipfel PF, Wallich R, Lea SM. 2005. A novel fold for the factor H-binding protein BbCRASP-1 of Borrelia burgdorferi. Nat Struct Mol Biol 12:276–277. doi:10.1038/nsmb902 15711564

[21] Siegel C, Schreiber J, Haupt K, Skerka C, Brade V, Simon MM, Stevenson B, Wallich R, Zipfel PF, Kraiczy P. 2008. Deciphering the ligand-binding sites in the Borrelia burgdorferi complement regulator-acquiring surface protein 2 (BbCRASP-2) required for interactions with the human immune regulators factor H and factor H-like protein 1. J Biol Chem 283:34855–34863. doi:10.1074/jbc.M805844200 18824548 PMC2596382

[22] Meri T, Amdahl H, Lehtinen MJ, Hyvärinen S, McDowell JV, Bhattacharjee A, Meri S, Marconi R, Goldman A, Jokiranta TS. 2013. Microbes bind complement inhibitor factor H via a common site. PLoS Pathog 9:e1003308. doi:10.1371/journal.ppat.1003308 23637600 PMC3630169

[23] Mühleip JJ, Lin Y-P, Kraiczy P. 2018. Further insights into the interaction of human and animal complement regulator factor H with viable Lyme disease spirochetes. Front Vet Sci 5:346. doi:10.3389/fvets.2018.00346 30766876 PMC6365980

[24] Rhodes DVL, Earnhart CG, Mather TN, Meeus PFM, Marconi RT. 2013. Identification of Borrelia burgdorferi ospC genotypes in canine tissue following tick infestation: Implications for Lyme disease vaccine and diagnostic assay design. Vet J 198:412–418. doi:10.1016/j.tvjl.2013.07.019 23962611 PMC3872846

[25] McDowell JV, Wolfgang J, Tran E, Metts MS, Hamilton D, Marconi RT. 2003. Comprehensive analysis of the factor H binding capabilities of Borrelia species associated with lyme disease: delineation of two distinct classes of factor H binding proteins. Infect Immun 71:3597–3602. doi:10.1128/IAI.71.6.3597-3602.2003 12761145 PMC155754

[26] Kimpston CN, Hatke AL, Castelli B, Otto N, Tiffin HS, Machtinger ET, Brown JD, Van Why KR, Marconi RT. 2022. High prevalence of antibodies against canine parvovirus and canine distemper virus among coyotes and foxes from Pennsylvania: implications for the intersection of companion animals and wildlife. Microbiol Spectr 10:e0253221. doi:10.1128/spectrum.02532-21 35080421 PMC8791182

[27] Horn EJ, Dempsey G, Schotthoefer AM, Prisco UL, McArdle M, Gervasi SS, Golightly M, De Luca C, Evans M, Pritt BS, Theel ES, Iyer R, Liveris D, Wang G, Goldstein D, Schwartz I. 2020. The lyme disease biobank - characterization of 550 patient and control samples from the east coast and upper midwest of the United States. J Clin Microbiol 58:e00032-20. doi:10.1128/JCM.00032-20 32102853 PMC7269379

[28] Kraiczy P, Hellwage J, Skerka C, Kirschfink M, Brade V, Zipfel PF, Wallich R. 2003. Immune evasion of Borrelia burgdorferi: mapping of a complement-inhibitor factor H-binding site of BbCRASP-3, a novel member of the Erp protein family. Eur J Immunol 33:697–707. doi:10.1002/eji.200323571 12616490

[29] Stevenson B, Bono JL, Schwan TG, Rosa P. 1998. Borrelia burgdorferi Erp proteins are immunogenic in mammals infected by tick bite, and their synthesis is inducible in cultured bacteria. Infect Immun 66:2648–2654. doi:10.1128/IAI.66.6.2648-2654.1998 9596729 PMC108251

[30] Akins DR, Caimano MJ, Yang X, Cerna F, Norgard MV, Radolf JD. 1999. Molecular and evolutionary analysis of Borrelia burgdorferi 297 circular plasmid-encoded lipoproteins with OspE- and OspF-like leader peptides. Infect Immun 67:1526–1532. doi:10.1128/IAI.67.3.1526-1532.1999 10024606 PMC96492

[31] Marconi RT, Sung SY, Hughes CA, Carlyon JA. 1996. Molecular and evolutionary analyses of a variable series of genes in Borrelia burgdorferi that are related to ospE and ospF, constitute a gene family, and share a common upstream homology box. J Bacteriol 178:5615–5626. doi:10.1128/jb.178.19.5615-5626.1996 8824605 PMC178399

[32] McDowell JV, Sung SY, Price G, Marconi RT. 2001. Demonstration of the genetic stability and temporal expression of select members of the lyme disease spirochete OspF protein family during infection in mice. Infect Immun 69:4831–4838. doi:10.1128/IAI.69.8.4831-4838.2001 11447157 PMC98571

[33] Hovis KM, McDowell JV, Griffin L, Marconi RT. 2004. Identification and characterization of a linear-plasmid-encoded factor H-binding protein (FhbA) of the relapsing fever spirochete Borrelia hermsii. J Bacteriol 186:2612–2618. doi:10.1128/JB.186.9.2612-2618.2004 15090501 PMC387808

[34] McDowell JV, Harlin ME, Rogers EA, Marconi RT. 2005. Putative coiled-coil structural elements of the BBA68 protein of Lyme disease spirochetes are required for formation of its factor H binding site. J Bacteriol 187:1317–1323. doi:10.1128/JB.187.4.1317-1323.2005 15687195 PMC545637

[35] Hovis KM, Schriefer ME, Bahlani S, Marconi RT. 2006. Immunological and molecular analyses of the Borrelia hermsii factor H and factor H-like protein 1 binding protein, FhbA: demonstration of its utility as a diagnostic marker and epidemiological tool for tick-borne relapsing fever. Infect Immun 74:4519–4529. doi:10.1128/IAI.00377-06 16861638 PMC1539583

[36] Hovis KM, Tran E, Sundy CM, Buckles E, McDowell JV, Marconi RT. 2006. Selective binding of Borrelia burgdorferi OspE paralogs to factor H and serum proteins from diverse animals: possible expansion of the role of OspE in Lyme disease pathogenesis. Infect Immun 74:1967–1972. doi:10.1128/IAI.74.3.1967-1972.2006 16495576 PMC1418677

[37] Schuler E, Marconi RT. 2021. The Leptospiral General Secretory Protein D (GspD), a secretin, elicits complement-independent bactericidal antibody against diverse Leptospira species and serovars. Vaccine X 7:100089. doi:10.1016/j.jvacx.2021.100089 33733085 PMC7941034

[38] Izac JR, O’Bier NS, Oliver LD, Camire AC, Earnhart CG, LeBlanc Rhodes DV, Young BF, Parnham SR, Davies C, Marconi RT. 2020. Development and optimization of OspC chimeritope vaccinogens for lyme disease. Vaccine 38:1915–1924. doi:10.1016/j.vaccine.2020.01.027 31959423 PMC7085410

[39] Earnhart CG, Rhodes DVL, Smith AA, Yang X, Tegels B, Carlyon JA, Pal U, Marconi RT. 2014. Assessment of the potential contribution of the highly conserved C-terminal motif (C10) of Borrelia burgdorferi outer surface protein C in transmission and infectivity. Pathog Dis 70:176–184. doi:10.1111/2049-632X.12119 24376161 PMC4497580

[40] Camire AC, O’Bier NS, Patel DT, Cramer NA, Straubinger RK, Breitschwerdt EB, Funk RA, Marconi RT. 2022. FtlA and FtlB are candidates for inclusion in a next-generation multiantigen subunit vaccine for Lyme disease. Infect Immun 90:e0036422. doi:10.1128/iai.00364-22 36102656 PMC9584329

[41] Marconi RT, Garcia-Tapia D, Hoevers J, Honsberger N, King VL, Ritter D, Schwahn DJ, Swearingin L, Weber A, Winkler MTC, Millership J. 2020. VANGUARD®crLyme: a next generation Lyme disease vaccine that prevents B. burgdorferi infection in dogs. Vaccine X 6:100079. doi:10.1016/j.jvacx.2020.100079 33336185 PMC7733144

[42] Tochilina AG, Belova IV, Ilyicheva TN, Marchenko VY, Zhirnov VA, Molodtsova SB, Ikonnikov AV, Muhkina IV, Blagonravova AS, Soloveva IV. 2022. Genome features of probiotic bifidobacteria determining their strain-specific properties. Sovrem Tekhnologii Med 14:36–43. doi:10.17691/stm2022.14.5.04 37181836 PMC10171061

[43] Huerta-Cepas J, Szklarczyk D, Forslund K, Cook H, Heller D, Walter MC, Rattei T, Mende DR, Sunagawa S, Kuhn M, Jensen LJ, von Mering C, Bork P. 2016. eggNOG 4.5: a hierarchical orthology framework with improved functional annotations for eukaryotic, prokaryotic and viral sequences. Nucleic Acids Res 44:D286–D293. doi:10.1093/nar/gkv1248 26582926 PMC4702882

[44] Page AJ, Cummins CA, Hunt M, Wong VK, Reuter S, Holden MTG, Fookes M, Falush D, Keane JA, Parkhill J. 2015. Roary: rapid large-scale prokaryote pan genome analysis. Bioinformatics 31:3691–3693. doi:10.1093/bioinformatics/btv421 26198102 PMC4817141

[45] Xu Y, Bruno JF, Luft BJ. 2003. Detection of genetic diversity in linear plasmids 28-3 and 36 in Borrelia burgdorferi sensu stricto isolates by subtractive hybridization. Microb Pathog 35:269–278. doi:10.1016/s0882-4010(03)00152-9 14580390

[46] Lemieux JE, Huang W, Hill N, Cerar T, Freimark L, Hernandez S, Luban M, Maraspin V, Bogovič P, Ogrinc K, Ruzič-Sabljič E, Lapierre P, Lasek-Nesselquist E, Singh N, Iyer R, Liveris D, Reed KD, Leong JM, Branda JA, Steere AC, Wormser GP, Strle F, Sabeti PC, Schwartz I, Strle K. 2023. Whole genome sequencing of human Borrelia burgdorferi isolates reveals linked blocks of accessory genome elements located on plasmids and associated with human dissemination. PLoS Pathog 19:e1011243. doi:10.1371/journal.ppat.1011243 37651316 PMC10470944

[47] Lemieux JE, Huang W, Hill N, Cerar T, Freimark L, Hernandez S, Luban M, Maraspin V, Bogovič P, Ogrinc K, Ruzič-Sabljič E, Lapierre P, Lasek-Nesselquist E, Singh N, Iyer R, Liveris D, Reed KD, Leong JM, Branda JA, Steere AC, Wormser GP, Strle F, Sabeti PC, Schwartz I, Strle K. 2023. Whole genome sequencing of human Borrelia burgdorferi isolates reveals linked blocks of accessory genome elements located on plasmids and associated with human dissemination. PLoS Pathog 19:e1011243. doi:10.1371/journal.ppat.1011243 37651316 PMC10470944

[48] Tokarz R, Anderton JM, Katona LI, Benach JL. 2004. Combined effects of blood and temperature shift on Borrelia burgdorferi gene expression as determined by whole genome. Infect Immun 72:5419–5432. doi:10.1128/IAI.72.9.5419-5432.2004 15322040 PMC517457

[49] Akins DR, Bourell KW, Caimano MJ, Norgard MV, Radolf JD. 1998. A new animal model for studying Lyme disease spirochetes in a mammalian host-adapted state. J Clin Invest 101:2240–2250. doi:10.1172/JCI2325 9593780 PMC508812

[50] McDowell JV, Hovis KM, Zhang H, Tran E, Lankford J, Marconi RT. 2006. Evidence that the BBA68 protein (BbCRASP-1) of the Lyme disease spirochetes does not contribute to factor H-mediated immune evasion in humans and other animals. Infect Immun 74:3030–3034. doi:10.1128/IAI.74.5.3030-3034.2006 16622245 PMC1459725

[51] Talagrand-Reboul E, Westermann B, Raess MA, Schnell G, Cantero P, Barthel C, Ehret-Sabatier L, Jaulhac B, Boulanger N. 2020. Proteomic as an exploratory approach to develop vaccines against tick-borne diseases using Lyme borreliosis as a test case. Vaccines (Basel) 8:463. doi:10.3390/vaccines8030463 32825641 PMC7564290

[52] Hart T, Nguyen NTT, Nowak NA, Zhang F, Linhardt RJ, Diuk-Wasser M, Ram S, Kraiczy P, Lin YP. 2018. Polymorphic factor H-binding activity of CspA protects Lyme borreliae from the host complement in feeding ticks to facilitate tick-to-host transmission. PLoS Pathog 14:e1007106. doi:10.1371/journal.ppat.1007106 29813137 PMC5993331

[53] Baum E, Grosenbaugh DA, Barbour AG. 2014. Diversity of antibody responses to Borrelia burgdorferi in experimentally infected beagle dogs. Clin Vaccine Immunol 21:838–846. doi:10.1128/CVI.00018-14 24695775 PMC4054242

[54] Dulebohn DP, Bestor A, Rosa PA. 2013. Borrelia burgdorferi linear plasmid 28-3 confers a selective advantage in an experimental mouse-tick infection model. Infect Immun 81:2986–2996. doi:10.1128/IAI.00219-13 23753630 PMC3719586

[55] Coleman AS, Yang X, Kumar M, Zhang X, Promnares K, Shroder D, Kenedy MR, Anderson JF, Akins DR, Pal U. 2008. Borrelia burgdorferi complement regulator-acquiring surface protein 2 does not contribute to complement resistance or host infectivity. PLoS One 3:3010e. doi:10.1371/journal.pone.0003010 PMC2526170 18714378

[56] Hellwage J, Meri T, Heikkilä T, Alitalo A, Panelius J, Lahdenne P, Seppälä IJ, Meri S. 2001. The complement regulator factor H binds to the surface protein OspE of Borrelia burgdorferi. J Biol Chem 276:8427–8435. doi:10.1074/jbc.M007994200 11113124

[57] Liang FT, Philipp MT. 1999. Analysis of antibody response to invariable regions of VlsE, the variable surface antigen of Borrelia burgdorferi. Infect Immun 67:6702–6706. doi:10.1128/IAI.67.12.6702-6706.1999 10569796 PMC97088

[58] Baum E, Hue F, Barbour AG. 2012. Experimental infections of the reservoir species Peromyscus leucopus with diverse strains of Borrelia burgdorferi, a Lyme disease agent. mBio 3:e00434-12. doi:10.1128/mBio.00434-12 23221801 PMC3517863

[59] Wachter J, Cheff B, Hillman C, Carracoi V, Dorward DW, Martens C, Barbian K, Nardone G, Renee Olano L, Kinnersley M, Secor PR, Rosa PA. 2023. Coupled induction of prophage and virulence factors during tick transmission of the Lyme disease spirochete. Nat Commun 14:198. doi:10.1038/s41467-023-35897-3 36639656 PMC9839762

